# Effect on Foot Width With Triplanar Tarsometatarsal Arthrodesis for Hallux Valgus

**DOI:** 10.1177/2473011420934804

**Published:** 2020-08-13

**Authors:** Justin Vaida, Justin J. Ray, Taylor L. Shackleford, William T. DeCarbo, Daniel J. Hatch, Paul Dayton, Jody P. McAleer, W. Bret Smith, Robert D. Santrock

**Affiliations:** 1 Department of Orthopaedics, 24041 West Virginia University, Morgantown, WV, USA; 2 St Clair Orthopedic Associates, Pittsburgh, PA, USA; 3 380410 Foot and Ankle Center of Northern Colorado, Greeley, CO, USA; 4 Foot & Ankle Center of Iowa, Midwest Bunion Center, Ankeny, IA, USA; 5 485122 Jefferson City Medical Group, Department of Podiatry, Jefferson City, MO, USA; 6 Mercy Orthopedic Associates, Mercy Regional Medical Center, Durango, CO, USA

**Keywords:** hallux valgus, foot width, modified Lapidus, first tarsometatarsal arthrodesis, bunion

## Abstract

**Background:**

Foot width reduction is a desirable cosmetic and functional outcome for patients with hallux valgus. Triplanar first tarsometatarsal (TMT) arthrodesis could achieve this goal by 3-dimensional correction of the deformity. The aim of this study was to evaluate changes in bony and soft tissue width in patients undergoing triplanar first TMT arthrodesis.

**Methods:**

After receiving Institutional Review Board approval, charts were retrospectively reviewed for patients undergoing triplanar first TMT arthrodesis for hallux valgus at 4 institutions between 2016 and 2019. Patients who underwent concomitant first metatarsal head osteotomies (eg, Silver or Chevron) or fifth metatarsal osteotomies were excluded. Preoperative and postoperative anteroposterior weightbearing radiographs were compared to evaluate for changes in bony and soft tissue width. One hundred forty-eight feet from 144 patients (48.1 ± 15.7 years, 92.5% female) met inclusion criteria.

**Results:**

Preoperative osseous foot width was 96.2 mm, compared to 85.8 mm postoperatively (*P* < .001). Preoperative soft tissue width was 106.6 mm, compared to 99.3 mm postoperatively (*P* < .001). Postoperatively, patients had an average 10.4 ± 4.0 mm reduction (10.8% reduction) in osseous width and average 7.3 ± 4.0 mm reduction (6.8% reduction) in soft tissue width.

**Conclusions:**

Triplanar first TMT arthrodesis reduced both osseous and soft tissue foot width, providing a desirable cosmetic and functional outcome for patients with hallux valgus. Future studies are needed to determine if patient satisfaction and outcome measures correlate with reductions in foot width. **Level of evidence**: Level III, retrospective comparative study

## Introduction

Foot width reduction is a desirable cosmetic and functional outcome for patients with hallux valgus. Patients with hallux valgus typically present with pain along the medial prominence at the first metatarsophalangeal (MTP) joint. There is often associated difficulty with certain types of footwear, especially constricting footwear, and symptoms frequently persist despite shoe modifications. Although cosmetics or foot appearance alone is not an indication for surgery, patient satisfaction after hallux valgus surgery is significantly associated with improvement in footwear problems after surgery.^11^

Few studies have evaluated changes in foot width after operative correction for hallux valgus; the majority of previous studies evaluated changes in foot width after metatarsal osteotomies with varying results.^4,7,13^ A recent study by Conti et al^2^ found that a modified Lapidus in combination with a modified McBride (plus medial eminence resection) and Akin osteotomy resulted in significant decreases in bony foot width (mean 8.9 mm; 9.1% reduction) and soft tissue width (mean 6.9 mm; 6.3% reduction). The study by Conti et al measured foot width changes after a modified Lapidus procedure using a crossed-screw technique, with patients made nonweightbearing for at least 6 weeks after surgery.^2^ No previous study has looked at changes in foot width after a triplanar first tarsometatarsal (TMT) arthrodesis (without medial eminence resection) via a locked plating technique with early weightbearing.

The authors believe that a triplanar first TMT arthrodesis is optimal to achieve hallux valgus correction in all 3 planes. The purpose of our study was to evaluate changes in bony and soft tissue width in patients undergoing triplanar TMT arthrodesis for hallux valgus. It was our hypothesis that triplanar TMT arthrodesis would result in significant decreases in bony and soft tissue width as measured on weightbearing anteroposterior (AP) radiographs.

## Methods

### Patient Population

This study was a retrospective chart review of patients undergoing triplanar first TMT arthrodesis (Lapiplasty System, Treace Medical Concepts, Inc, Ponte Vedra, FL) for hallux valgus correction at 4 institutions between 2016 and 2019. The study received Institutional Review Board approval at the host institution, in addition to research agreements with the additional sites. A foot and ankle fellowship–trained orthopedic surgeon (1 site) or podiatrist (3 sites) performed all surgeries. Basic demographic data including age, sex, body mass index (BMI), smoking status, and presence of diabetes were collected.

Inclusion criteria included 144 patients (148 feet) aged 48.1 ± 15.7 years (92.5% female) with symptomatic hallux valgus who underwent triplanar TMT arthrodesis by 4 surgeons with a minimum follow-up of 5 months. Patients who underwent concomitant first metatarsal head osteotomies (eg, Silver or Chevron) or fifth metatarsal osteotomies were excluded. Patients with previous surgery of the first ray were also excluded. Demographic characteristics are shown in Table 1. There were 8 diabetics (5.4%) and 12 current smokers (8.1%) included in the study. The average BMI was 27.9. In terms of additional procedures, 145 patients (98.0%) had a modified McBride (without traditional medial eminence resection), whereas 20 patients (13.5%) had an additional Akin osteotomy. An additional screw for intercuneiform instability was required in 61 patients (41.2%).

**Table 1.** Patient Demographics.

Characteristic	Study Population
Age, mean ± SD, y	48.1 ± 15.7
Gender, n/N (%)	
Female	137/148 (92.5)
Male	11/148 (7.5)
Body mass index, mean ± SD	27.9 ± 5.7
Smoking status, n/N (%)	
None	136/148 (91.9)
Current	12/148 (8.1)
Diabetic, n/N (%)	8/148 (5.4)
Additional procedures, n/N (%)	
Modified McBride	145/148 (98.0)
Akin osteotomy	20/148 (13.5)

### Radiographic Assessment

**Figure 1.**
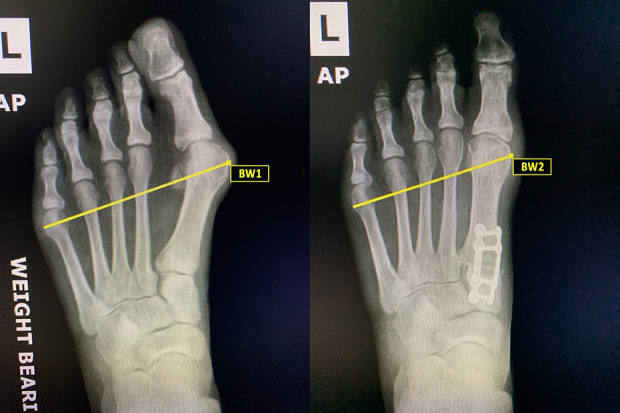
Weightbearing radiographs showing changes in bony width preoperative (BW1) and postoperative (BW2) after triplanar first tarsometatarsal arthrodesis. Bony width was defined as the distance from the most medial aspect of the first metatarsal head to the most lateral aspect of the fifth metatarsal head.

**Figure 2.**
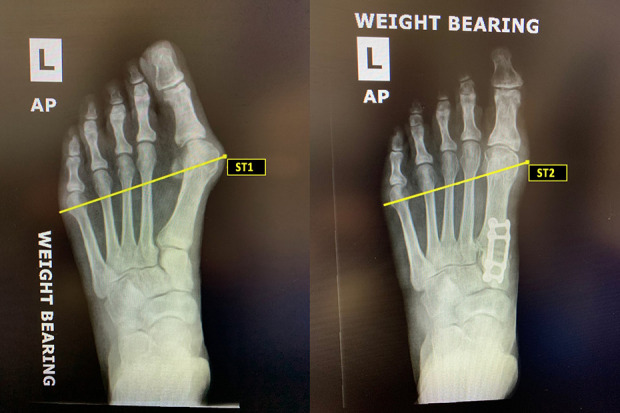
Weightbearing radiographs showing changes in soft tissue foot width preoperative (ST1) and postoperative (ST2) after triplanar first tarsometatarsal arthrodesis. Soft tissue width was defined as the distance from the most medial soft tissue overlying the first metatarsal head to the most lateral soft tissue overlying the fifth metatarsal head.

Weightbearing AP radiographs obtained preoperatively and at 5 months postoperatively were measured for changes in bony and soft tissue width. Bony width was defined as the distance from the most medial aspect of the first metatarsal head to the most lateral aspect of the fifth metatarsal head (Figure 1). Soft tissue width was defined as the distance from the most medial soft tissue overlying the first metatarsal head to the most lateral soft tissue overlying the fifth metatarsal head (Figure 2). A single reviewer blinded to patient outcome reviewed all radiographs at each respective site. Radiographic measures were conducted using Synapse Radiology PACS (Synapse PACS, version 5.5.002, FUJIFILM Medical Systems, USA, Inc). Although weightbearing radiographs were not calibrated, the images were taken by experienced radiology technicians in a consistent manner between institutions.

### Operative Technique

The detailed operative technique has been previously published.^9^ In summary, a dorsal longitudinal incision was centered over the first TMT joint. The first TMT joint was exposed dorsally and medially via subperiosteal dissection, followed by a plantar release of the joint. Next, a custom fulcrum and positioner device was used to achieve triplanar deformity correction (axial, transverse, sagittal). Fluoroscopy was used to confirm deformity correction. Placing a Kirschner wire through the positioner temporarily held the position. A joint-seeker device was then placed in the first TMT joint, followed by the cutting guide to make the bony cuts on the medial cuneiform and base of the first metatarsal. After fenestration of the joint surfaces, the first TMT joint was axially compressed. Final fixation was achieved via a biplanar technique involving 2 low-profile 4-hole titanium locking plates applied in 90-90 fashion, with one plate applied dorsally and the other plate applied medially across the first TMT joint. The surgeon had the option to place an additional screw if there was evidence of intercuneiform instability. The incision was then closed in the standard fashion, followed by placement of a sterile dressing and walking boot.

### Postoperative Protocol

In general, patients were permitted early full weightbearing in a walking boot on the day of surgery. Weightbearing was delayed for 2 weeks at one institution. Weightbearing AP radiographs of the operative foot were taken at 5 months postoperatively and compared to preoperative measurements.

### Statistical Analysis

Mean and standard deviation were reported for continuous variables, in addition to percentages and ranges where appropriate. Postoperative bony and soft tissue widths were compared to preoperative values using independent Student *t* tests. One-way analysis of variance was used to evaluate for significant differences in bony and soft tissue foot width based on demographic characteristics. JMP 14.1 (SAS Institute Inc, Cary, NC) was used for statistical analysis. A *P* value of <.05 was considered statistically significant.

## Results

Measurements of preoperative and postoperative bony and soft tissue width are shown in Table 2. All 148 feet demonstrated a decrease in bony foot width after surgery. The mean preoperative bony foot width was 96.2 ± 7.2 mm (range, 76.5-115.8 mm), compared with a mean postoperative bony foot width of 85.8 ± 6.9 mm (range, 69.0-103.6 mm; *P* < .001). After triplanar first tarsometatarsal arthrodesis, bony foot width decreased significantly by an average of 10.4 ± 4.0 mm (10.8% reduction; range, 0.1-21.8 mm) compared with preoperative values (*P* < .001).

**Table 2.** Average Foot Width Measures after Triplanar TMT Arthrodesis Compared to Preoperative.

	Preoperative, Mean ± SD	Postoperative, Mean ± SD	Change in Width, Mean ± SD	*P* Value
Bony width, mm	96.2 ± 7.2	85.8 ± 6.9	10.4 ± 4.0	<.001
Soft tissue width, mm	106.6 ± 7.4	99.3 ± 7.3	7.3 ± 4.0	<.001

Abbreviation: TMT, tarsometatarsal.

All 148 feet also demonstrated a decrease in soft tissue foot width after surgery. The mean preoperative soft tissue width was 106.6 ± 7.4 mm (range, 86.1-128.4 mm). After surgery, the postoperative soft tissue width decreased to 99.3 ± 7.3 mm (range, 82.8-120.0 mm). Postoperatively, patients experienced a significant decrease in soft tissue width by an average of 7.3 ± 4.0 mm (6.8% reduction; range, 0.0-19.4 mm) compared to preoperative numbers (*P* < .001).

Bony width changes were significantly higher in males (mean 12.7 ± 1.2 mm) compared to females (mean 10.2 ± 0.3 mm; *P* = .049). Soft tissue width was not significantly different between males and females (*P* = .76). There was no significant difference in bony or soft tissue foot width based on the addition of a modified McBride procedure (*P* = .91 and *P* = .23, respectively) or an Akin osteotomy (*P* = .12 and *P* = .38, respectively). There was no significant difference in bony foot width based on age, BMI, diabetes, or smoking (*P* = .35, .40, .30, .60, respectively). Similarly, no significant difference existed in the mean soft tissue foot width based on age, BMI, diabetes, or smoking (*P* = .27, .25, .76, .44, respectively).

## Discussion

Overall, our study found significant decreases in bony and soft tissue widths after triplanar first tarsometatarsal arthrodesis. Bony width decreased by 10.4 mm (10.8%) postoperatively, whereas soft tissue width decreased 7.3 mm (6.8%) postoperatively after triplanar first TMT arthrodesis. The majority of the patients in our study had a concomitant modified McBride procedure (98.0%), whereas only 20 patients (13.5%) had an Akin osteotomy. None of the patients in our study had a traditional medial eminence resection as part of the modified McBride procedure. The results support our hypothesis that a triplanar first TMT arthrodesis would result in significant decreases in bony and soft tissue widths postoperatively.

The reductions in bony and soft tissue foot width in our study are similar to a recent study by Conti et al that measured reductions in bony and soft tissue widths after modified Lapidus arthrodesis with a modified McBride and Akin osteotomy.^2^ In our study, bony foot width decreased by 10.4 mm (10.8%) compared with 8.9 mm (9.1%) on radiographs in the Conti et al study. Soft tissue width decreased by 7.3 mm (6.8%) in our study compared with 6.9 mm (6.3%) on radiographs in the Conti et al study. A notable strength of their study is that bony and soft tissue width measurements were also taken and confirmed using weightbearing computed tomography (WBCT) scans. They found similar numbers using WBCT scans, noting decreases in bony foot width of 7.9 mm (8.4%) and decreases in soft tissue foot width of 6.7 mm (6.4%). The Conti et al study used a crossed compression screw technique for the tarsometatarsal arthrodesis in comparison to the biplanar 90-90 locking plates used for triplanar first TMT arthrodesis in our study.^2^ Both techniques used for first tarsometatarsal arthrodesis achieved significant and similar reductions in bony and soft tissue foot widths.

There are several notable differences between the Conti et al study and our study.^2^ The study population in our study was much larger (148 feet in 148 patients) in comparison to the Conti et al study (31 feet in 30 patients). In their study, patients underwent a modified Lapidus arthrodesis (crossed compression screws) in combination with a modified McBride (100% of patients) and an Akin osteotomy (80.6% of patients). In addition, all patients in their study underwent a medial eminence resection as part of the modified McBride with an average decrease of 1.8 mm in first metatarsal head size on WBCT scans, which may have confounded the reductions in bony and soft tissue width.^2^ In our study, none of the patients underwent a medial eminence resection and only 20% of patients received an Akin osteotomy. This difference may indicate that the triplanar first TMT arthrodesis technique used in our study more reliably and consistently achieved a reduction in bony and soft tissue width without the need for additional procedures.

Previous studies measuring foot width changes after first metatarsal osteotomies have reported varying results. A study by Jung et al found average decreases in bony width of 16 mm (16%) in 117 patients after a proximal chevron metatarsal osteotomy in combination with a distal soft tissue procedure, Akin osteotomy, and medial eminence resection.^4^ Soft tissue foot width was not measured in the Jung et al study,^4^ and the decreases in foot width were likely confounded by the large medial eminence resection. Tenenbaum et al^13^ noted 5% decreases in bony foot width and 2% decreases in soft tissue foot width in 71 patients treated with a scarf osteotomy in combination with an Akin osteotomy and distal soft tissue release. However, the study by Tenenbaum et al^13^ did not quantify the foot width decreases in millimeters. Although foot width changes after metatarsal osteotomies have been variable and inconsistent, our study and recent evidence demonstrate that the first tarsometatarsal arthrodesis more reliably and consistently leads to reductions in foot width.^2^

In our study, patients were permitted early weightbearing in a walking boot. Although one surgeon delayed weightbearing for 2 weeks, the majority of patients were allowed full weightbearing in a walking boot on the day of surgery. A notable difference in the study by Conti et al^2^ is that patients were made nonweightbearing for at least 6 weeks after surgery until evidence of first TMT fusion site healing on radiographs and clinical examination. Several recent studies have demonstrated low nonunion rates and maintained correction after a first TMT arthrodesis, even with early weightbearing.^1,5,6,8,9^ It is important to mobilize patients early after surgery to avoid the complications of prolonged immobilization. The results of our study showed that significant reductions in bony and soft tissue foot widths may still be achieved after a triplanar first TMT arthrodesis with early weightbearing.

Reduction of foot width is an important outcome after hallux valgus correction, especially as it pertains to the improved ability to wear shoes. In addition to reduced pain, patient satisfaction after hallux valgus surgery is inextricably linked to foot appearance and the ability to wear shoes.^3,10-12^ A painless great toe, correction of footwear problems, and improved walking ability were the most important factors influencing outcomes in a study of more than 200 patients undergoing operative correction for hallux valgus.^11^ In a prospective study of 95 women undergoing hallux valgus surgery, Dawson et al^3^ found that foot appearance and the ability to wear a range of shoes after surgery were critical to patient satisfaction. Tai et al^12^ similarly found that the most important patient expectations after hallux valgus surgery were improved walking, reduced pain, and wearing daily shoes. Further, Saro et al^10^ concluded that the ability to wear preferred footwear was more important to quality of life following hallux valgus surgery than either hallux valgus angle (HVA) or intermetatarsal angle (IMA) correction. Although attention should be paid to radiographic parameters such as HVA or IMA, patient satisfaction remains inextricably tied to perception of correction as it relates to overall foot appearance and the ability to select footwear.

Limitations of this study included those inherent for any retrospective multicenter study design. There was slight surgeon variability regarding operative technique and postoperative protocol, although these differences also make the results more generalizable. Another limitation was the possibility of measurement bias, as each site had a single reviewer blinded to patient outcome measure preoperative and postoperative bony and soft tissue widths according to study protocol. The inability to correlate bony and soft tissue width changes to radiographic measures and patient satisfaction was a limitation that should be addressed in future studies. The lack of calibrated radiographs and inability to measure foot width changes using weightbearing CT scans was another limitation.

There are several notable strengths of this study. Although a recent study investigated foot width changes after a first TMT arthrodesis, our study was the first to measure foot width changes after a first TMT arthrodesis using a different operative technique with early weightbearing.^2^ The sample size in our study was much larger than previous studies measuring foot width changes after hallux valgus correction. This was a multicenter study with 4 different surgeons using a single fixation construct with a similar operative technique, making the results more generalizable and more reproducible. Future studies to measure foot width changes with WBCT scan and correlate these findings with radiographic changes and patient satisfaction after triplanar first tarsometatarsal arthrodesis are currently under way.

In conclusion, triplanar first tarsometatarsal arthrodesis resulted in significant decreases in both soft tissue and bony foot width providing a desirable cosmetic and functional outcome for patients with hallux valgus. Future studies are needed to determine if patient satisfaction and outcome measures correlate with reductions in foot width.

## Supplemental Material

Supplemental Material, FAO934804-ICMJE - Effect on Foot Width With Triplanar Tarsometatarsal Arthrodesis for Hallux Valgus Click here for additional data file. Supplemental Material, FAO934804-ICMJE for Effect on Foot Width With Triplanar Tarsometatarsal Arthrodesis for Hallux Valgus by Justin Vaida, Justin J. Ray, Taylor L. Shackleford, William T. DeCarbo, Daniel J. Hatch, Paul Dayton, Jody P. McAleer, W. Bret Smith and Robert D. Santrock in Foot & Ankle Orthopaedics
